# The Influence of Metal Lithium and Alkyl Chain in the Nucleating Agent Lauroyloxy-Substituted Aryl Aluminum Phosphate on the Crystallization and Optical Properties for iPP

**DOI:** 10.3390/polym14173637

**Published:** 2022-09-02

**Authors:** Fuhua Lin, Mi Zhang, Shuangdan Mao, Jianjun Zhang, Kezhi Wang, Jun Luo, Xinde Chen, Bo Wang, Yinghui Wei

**Affiliations:** 1 School of Materials Science and Engineering, Taiyuan University of Science and Technology, Taiyuan 030024, China; 2 Shanxi Provincial Institute of Chemical Industry (Co., Ltd.), Jinzhong 030600, China; 3 Key Laboratory of Renewable Energy, Guangzhou Institute of Energy Conversion, Chinese Academy of Sciences, Guangzhou 510640, China; 4 School of Chemical Engineering and Technology, Taiyuan University of Science and Technology, Taiyuan 030024, China; 5 Guangzhou Fibre Product Testing and Research Institute, Guangzhou 510220, China

**Keywords:** isotactic polypropylene (iPP), aryl phosphate salt, crystallization behavior, optical property, mechanical property

## Abstract

In this work, a kind of aryl phosphate salt nucleating agent (APAl-12C) was synthesized, which was replaced in the hydroxyl group on the aluminum hydroxy bis [2,2′-methylene-bis(4,6-di-tert-butylphenyl) phosphate] (APAl-OH) by lauroyloxy, which could improve the dispersion between the nucleating agent and the iPP matrix and reduce the migration potential of the nucleating agent in the iPP matrix by increasing the molecular weight. The structure of the nucleating agent APAl-12C was analyzed by fourier infrared spectroscopy (FT-IR ) and ^1^H NMR. The differential scanning calorimeter (DSC) results indicated that the addition of APAl-OH or APAl-12C alone was inferior to the commercial nucleating agent NA-21 (compounds of APAl-OH and Lithium laurate) in terms of the crystallization behavior, which may be due to the importance of metal Li in the crystallization property. Thus, the iPP/A12C-Li composites were prepared with APAl-12C, lithium laurate (lilaurate) and the iPP matrix. The crystallization behavior, morphology, optical and mechanical properties for the iPP/A12C-Li composites were systematically studied and compared with that of the iPP/NA-21 composite. Among the iPP/A12C-Li composites with the addition of 0.5 wt%, APAl-12C/Lilaurate had the fastest crystallization rate and reduced the haze value of the neat iPP from 36.03% to 9.89% without changing the clarity, which was better than that of the iPP/NA-21 composite. This was due to the weakening of the polarity of the APAl-12C after lauroyloxy substitution and better dispersion in the iPP matrix, resulting in a significant improvement in the optical properties.

## 1. Introduction

Isotactic polypropylene (iPP) has one of the best heat resistances among general resins and is widely used in the chemical industry, construction, automobile manufacturing and packaging materials because of its low density, easy processing, impact resistance and bending resistance [1,2,3]. Despite the outstanding advantages, the processing and application properties of iPP are directly affected by its crystallization speed, morphology and spherulite size, which restricts the application to a certain extent [4,5,6]. At present, the most simple and efficient modification method is adding the nucleating agent into the iPP matrix.

Aryl phosphate salts (APs) are a kind of important nucleating agent in the application of iPP modification [7]. The main commercial brands are NA-11, NA-21, TMP-6, etc. NA-21 is the third generation nucleating agent of APs, which has the advantages of stiffness enhancement, high transmittance, good thermal stability and no peculiar smell during processing. Especially for the optical performance of iPP, NA-21 is much superior to that of NA-11 [8].

**Figure 1:**
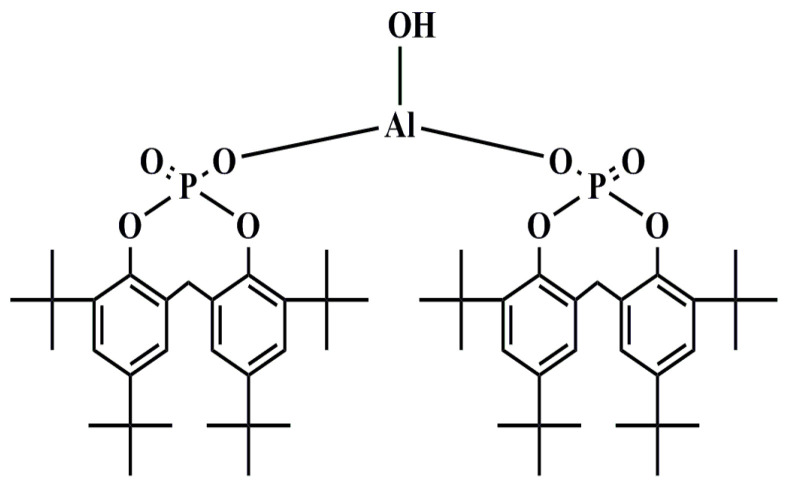
The chemical structure of APAl-OH.

As we all know, NA-21 is a kind of compound nucleating agent where the major constituent is aluminum hydroxy bis [2,2′-methylene-bis(4,6-di-tert-butylphenyl) phosphate] (APAl-OH, as shown in Figure 1), and the ligand is an alkyl carboxylic salt [9,10]. Several researchers have theoretically investigated that adding one component alone does not significantly improve the crystallization and the optical performance of iPP [11,12]. Kimura et al. [13] proposed, for the first time, that the synergistic effect of APAl-OH and lithium laurate can reduce the haze of PP from 32% to 5%. As a kind of additive nucleating agent, NA-21 is difficult to disperse in the iPP system because of the presence of strong polar phosphate compounds in its components [14].

At present, many studies have been devoted to improving the dispersion of the APs nucleating agents in the iPP matrix [15,16,17]. Li et al. pointed out that the NA-11 could be dispersed to the nanoscale by supercritical carbon dioxide (scCO_2_). The crystallization rate and mechanical properties of the PP composites with NA-11 dispersed by scCO_2_ were higher than those of untreated PP, while the trend of the haze and the spherulite size was just the opposite [18]. It has been proven that solvents can also promote the dispersion of nucleating agents [19]. The ethanol was chosen as a solvent to prepare micro emulsion lotion of the NA-11 and sodium benzoate composite nucleating agent. The size of the dispersed phase in PP is smaller, and the stiffness of the PP is increased obviously [20]. Meng et al. increased the dispersion of the NA-11 in the iPP by adding zeolite containing hydrogen bonds, accelerating the crystallization rate and improving the tensile strength and the flexural modulus [21]. These studies have suggested that reducing the size or dispersion of the nucleating agents by physical addition can improve the nucleation efficiency. However, the dispersion of the nucleating agent cannot be fundamentally solved, and the introduction of other substances may be affected by the nucleation ability of polymers [22]. In response to the problems, some researchers have theoretically investigated the modification of groups for the structure of the nucleating agents. Zhang et al. modified the glycerol ester onto a phosphate ester. The results indicated that the derivative made the crystallization temperature increase by 11 °C for iPP and the mechanical properties were significantly improved [23]. Long et al. introduced amino functional groups into phosphate ester to prepare a low melting point nucleating agent. Compared with the NA-11, the derivative particles were evenly dispersed in the iPP matrix and their compatibility with the iPP matrix was better than that of the NA-11 [24]. Despite the recent progress reviewed in modified NA-11, there are very few studies on the group modification of NA-21.

In this study, a new nucleating agent, APAl-12C, was synthesized by substituting alkylation for a hydroxyl group of APAl-OH. The primary goal of this research was to discuss the nucleation activities of the APAl-12C, NA-21 and APAl-12C/Lilaurate, focusing on their crystallization behavior and application properties in iPP, especially their optical and mechanical properties. Moreover, the increase in molecular weight of the nucleating agent has an important influence on reducing the migration potential in the iPP matrix, thereby lowering the impact on the environment. The nucleation behavior was preliminarily explored by a polarizing microscope (POM) and differential scanning calorimetry (DSC). It was expected to explore the role of the alkyl carboxylic acid functional group and Li metal in the nucleation process.

## 2. Materials and Methods

### 2.1. Materials

Isotactic polypropylene (K1012, Isotacticity: 97%, M_w_: 80,000–150,000) with a melt flow of 12 g/10 min was received from Sinopec Beijing Yanshan Company (Beijing, China). Aluminum isopropoxide, lauric acid and lithium laurate (lilaurate) were purchased from Shanghai McLean Biochemical Technology, China (Co., Ltd.). 2,2′-methylene bis(4,6-di-tert-butyl phenoxy). Phosphate and NA-21 nucleating agent were provided by Shanxi Provincial Institute of Chemical Industry, Jinzhong, China (Co., Ltd.). Toluene was provided from Shanghai Macklin Biochemical, Shanghai, China (Co., Ltd.). All reagents were all of analytical grade.

### 2.2. Synthesis of Nucleating Agent APAl-12C

**Figure 2:**
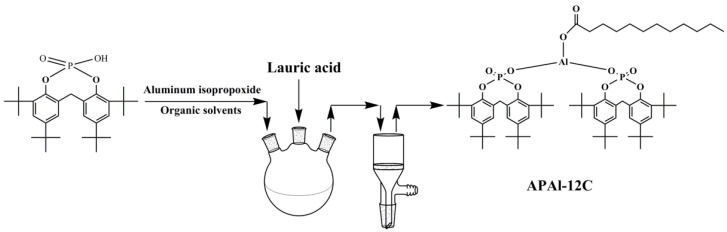
The synthesis of the APAl-12C.

The APAl-12C was synthesized as depicted in Figure 2. The addition of 2,2′-methylene bis(4,6-di-tert-butyl phenoxy) phosphate, aluminum isopropoxide and toluene to the reaction flask was followed by stirring and heating for 2 h. Then lauric acid was added to the mixture and maintained for 1 h. The nucleating agent APAl-12C was collected and washed three times using deionized water, and then dried in an oven at 105 °C for 24 h.

### 2.3. Preparation of the iPP Composites

The iPP composites were mixed together with different nucleating agents and extruded from a corotating twin-screw extruder. The barrel temperatures were maintained at 175, 180, 185 and 200 °C from the hopper to the mold, and the screw speed was set at 300 r/min. After pelletizing and drying, the extruded iPP composites were dried at above 100 °C for 12 h. The iPP composites with 0.3 wt%, APAl-OH, APAl-12C and NA-21, were prepared and abbreviated as iPP/AOH, iPP/A12C and iPP/NA-21, respectively.

### 2.4. Characterization

The Fourier transform infrared spectra (FT-IR) was recorded in the range of 4000–600 cm^−1^ using an FT-IR spectrophotometer (Perkin Elmer, Waltham, MA, USA) with the KBr pellet technique.

The nuclear magnetic resonance hydrogen spectrum (^1^H NMR) was recorded using a nuclear magnetic resonance spectrometer (Bruker Avance, Karlsruhe, Germany), operating at a frequency of 500 MHz for protons at room temperature. Deuterated methanol was used as the solvent to prepare solutions of 5 *w*/*v*% and the sweep width was 6 kHz.

The thermogravimetric analysis (TGA) of the samples was carried out by a TGA 1 (Mettler Toledo, Zurich, Switzerland) under a nitrogen flow rate of 60 mL/min from 25 to 800 °C with a heating rate of 10 °C/min.

The differential scanning calorimeter (DSC) was employed to observe the crystallization behavior of iPP composites by a DSC 1 (Mettler Toledo) under a nitrogen atmosphere. The conditions for the non-isothermal crystallization behavior of the iPP composites were as follows. The samples were first heated from 25 to 210 °C with a heating rate of 20 °C/min and maintained for 5 min to remove the thermal history. Then, the samples were cooled to 25 °C with a cooling rate of 10 °C/min. After cooling to 25 °C, the samples were reheated to 210 °C at a rate of 10 °C/min. The conditions for the isothermal crystallization behavior of the iPP composites were as follows. The samples were heated from 25 to 210 °C with a heating rate of 20 °C/min and maintained for 5 min to erase the thermal history. Then, the samples were cooled to 128 °C at a rate of 50 °C/min and kept at 128 °C for 1 h.

The crystallization morphology of the iPP composites was studied by a polarized optical microscope (POM, Leica DM 2700, Wetzlar, Germany) equipped with a hot-stage (Linkam THMS 600, Salfords, UK). The samples were heated at 230 °C for 5 min to erase any traces of crystals, and then rapidly cooled to 120 °C. The samples were kept at 120 °C until the crystallization process was completed.

The tensile strength test was carried out using a universal testing machine (M10, Instron, Norwood, MA, USA) with a crosshead speed of 5 mm/min, according to the GB/T1040.2-2006 standard, at a temperature of 25 ± 2 °C. The flexural modulus test was also carried out with the universal testing machine using a three-point bending test mode, according to the GB/T 9341-2008 standard, at a temperature of 25 ± 2 °C. The impact strength was carried out using an impact testing machine (GT-7045-HML, Gaotie, Taiwan, China) with a crosshead speed of 5 mm/min, according to the GB/T 1843-2008 standard, at a temperature of 25 ± 2 °C. At least five times for each sample were tested to obtain an averaged value with standard deviation.

The haze and clarity values of the iPP composites were tested using a WGT-2S test instrument (Shenguang, China).

The dispersion of the nucleating agents in the iPP matrix was characterized by a scanning electron microscope (SEM, JSM-6700F, JEOL, Tokyo, Japan). The test specimens were fractured under liquid nitrogen conditions until a smooth surface was obtained.

## 3. Results and Discussion

### 3.1. Characterization of the Nucleating Agent APAl-12C

**Figure 3:**
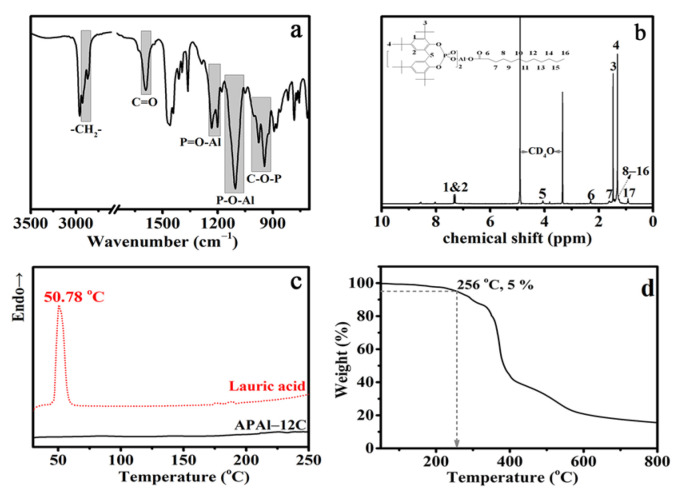
FT-IR (**a**), ^1^H NMR (**b**), DSC (**c**) and TGA (**d**) spectroscopy of the APAl-12C nucleating agent.

The APAl-12C is characterized by FT-IR, ^1^H NMR, DSC and TG spectroscopy, respectively. The structure of the nucleating agent APAl-12C is tested by FT-IR spectra in Figure 3a. The stretching vibration peaks of –CH_2_– and C=O are detected at about 2917 cm^−1^ and 1590 cm^−1^, which are attributed to the characteristic peaks of lauroyloxy [25]. Moreover, a strong peak at approximately 1476 cm^−1^ could be assigned to the benzene ring stretching vibration [26]. The peaks in the range of 1004 and 944 cm^−1^ are attributed to the stretching vibrations of C–O–P coming from 2,2′-methylene bis(4,6-di-tert-butyl phenoxy) phosphate [27]. In addition, the new peak centered at 1104 cm^−1^ corresponded to the stretching modes of the P–O–Al group from the synthesis reaction for the nucleating agent APAl-12C [28]. The FTIR shows the characteristic peaks of both lauric acid and 2,2′-methylene bis(4,6-di-tert-butyl phenoxy) phosphate, which was corroborated by the successful synthesis of the nucleating agent, APAl-12C. In order to further confirm the structure of the APAl-12C nucleating agent, the chemical shifts of hydrogen atoms at different positions in the molecular structure are analyzed by ^1^H NMR in Figure 3b. The protons of the benzene ring appear at 7.28 ppm and 7.33 ppm (“1&2”C–H), whereas the three methyl groups on tert butyl were adjacent to the benzene ring and appear at 1.46 ppm and at 1.31 ppm (“3”and “4”–CH_3_) [29,30]. The chemical shift of methylene connecting the two benzene rings appeared at 3.81 ppm (“5”–CH_2_). The methylene, which was adjacent to the carbon of the ester group, appeared at 2.30 ppm (“6”–CH_2_), and there were three splitting peaks in this peak, which related to its connection with another methylene [31]. The band of alkylic R–H (δH: 0.60–1.80 ppm) shows maxima at 0.9 ppm from terminal methyl hydrogens C–CH_3_ (“16”–CH_3_) and 1.3 ppm from polymethylene chains –(CH_2_)n– (“8–15”–CH_2_) [32]. The above description of the chemical shifts for APAl-12C is consistent with the reported literature. Therefore, the above-mentioned results of FT-IR and ^1^H NMR are in line with the successful synthesis of the nucleating agent APAl-12C.

As we all know, the thermostability of the nucleating agents is very important for commercial application [33]. The melting point of the synthetic APAl-12C is analyzed in Figure 3c. It can be seen from the DSC curves that there is no melting peak at 50.78 °C of APAl-12C compared with lauric acid. There is also no endothermic or exothermic process range from 25 to 250 °C in the APAl-12C curve. As shown in Figure 3d, in the TGA curve of the APAl-12C, the initial degradation temperature (defined as the temperature where 5% weight loss occurred [34]) is about 256 °C, which is higher than the 235 °C reported as the initial decomposition temperature of pure iPP [35]. Therefore, combined with the curves of DSC and TGA, it can be concluded that the APAl-12C nucleating agent has a thermostability below 250 °C, which will not change its structure during the processing of iPP.

### 3.2. Crystallization Behavior of the iPP Composites

**Figure 4:**
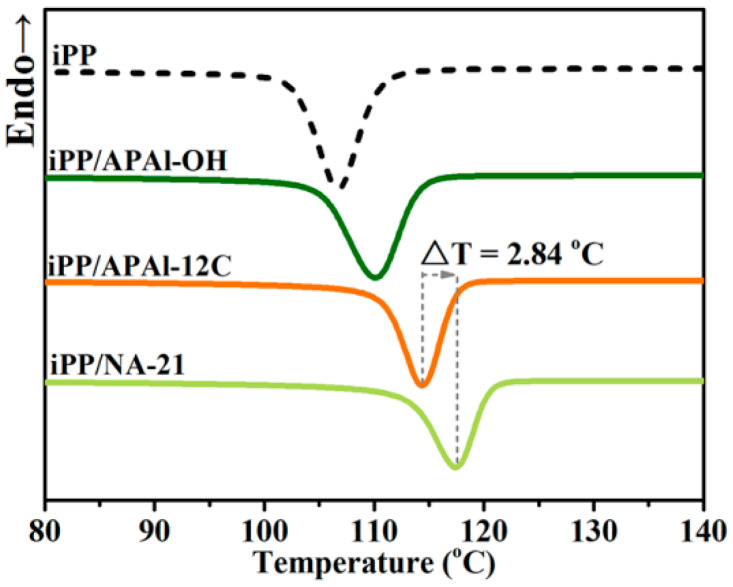
The DSC curves for the iPP composites.

The non-isothermal crystallization process is commonly used to study the nucleation ability of different nucleating agents for polymers [36]. The overall crystallization behavior of the iPP composites carried over in an N_2_ atmosphere, as shown in Figure 4. The neat iPP has a single crystallization peak at about 106.67 °C. The value of the crystallization peak temperatures (T_c,p_), both iPP/A12C and iPP/AOH, exceeded that of pure iPP, which revealed that the presence of APAl-OH or APAl-12C can shift the T_c,p_ of iPP towards a higher temperature regime slowly, promoting the iPP composites’ crystallization in advance during processing. It is worth mentioning that under the concentration of 0.3 wt% APAl-12C, the value of T_c,p_ for the iPP/A12C composite is 114.68 °C higher than 110.09 °C for the iPP/AOH composite with 0.3 wt% APAl-OH, suggesting that the nucleating ability of the APAl-12C is superior to APAl-OH, which is mainly ascribed to the decrease in polarity between APAl-12C and the iPP matrix because of the replacement of the hydroxyl group on the APAl-OH structure with lauroyloxy. On the other hand, the nucleation ability of the iPP/A12C composite is inferior to that of the iPP/NA-21 composite, where the value of T_c,p_ for the iPP/NA-21 composite is 2.84 °C higher than the iPP/AOH composite at the same concentration of the nucleating agents. The DSC result shows that the direct substitution of lauroyloxy structure can improve the nucleation ability of iPP to a certain extent, but it is far less than NA-21, which may be due to the role of metal Li in the NA-21 component, which is irreplaceable in terms of the crystallization behavior.

On the basis of this view that metal Li may be the key factor in the nucleation ability of iPP, we make the speculation that if metal Li is introduced into APAl-12C, will the crystallization property of iPP be improved to the equivalent of that of NA-21? So, the iPP/A12C-Li composites were prepared with the APAl-12C and lilaurate according to the formula in Table 1.

### 3.3. The Crystallization Behavior for the iPP/A12C-Li Composites

**Figure 5:**
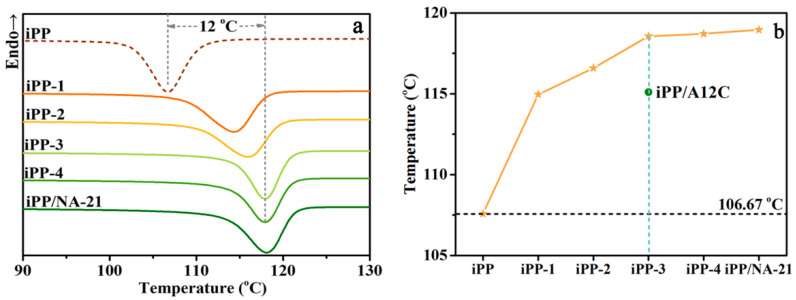
The non-isothermal crystallization curves (**a**) and the related *T*_c,p_ (**b**) for the iPP/A12C-Li composites under a cooling rate of 10 °C/min.

The overall non-isothermal crystallization behavior for the iPP/A12C-Li composites is carried over in an N_2_ atmosphere, as shown in Figure 5a, and the corresponding *T*_c,p_ values are presented in Figure 5b. The *T*_c,p_ for the iPP-3 composite with the concentration of 0.3 wt% APAl-12C/Lilaurate is increased to 117.86 °C compared with 114.68 °C of the iPP/A12C composite with 0.3 wt% of APAl-12C, further verifying that the presence of metal Li has an important influence on the nucleation ability of iPP. Meanwhile, with the increase in APAl-12C/Lilaurate concentration, the *T*_c,p_ for the iPP/A12C-Li composites gradually moved towards higher temperatures. While adding 0.5 wt% APAl-12C/lilaurate, the value of *T*_c,p_ for the iPP-4 composite reached 118.67 °C, which is 12 °C higher than pure iPP, illustrating the high synergistic nucleation efficiency between APAl-12C and lilaurate. Apart from this, there is no obvious difference between the APAl-12C/lilaurate and NA-21 as the aspect of *T*_c,p_, which can be verified that the crystallization property of the iPP composites with the APAl-12C/lilaurate is equivalent to that of the NA-21.

**Figure 6:**
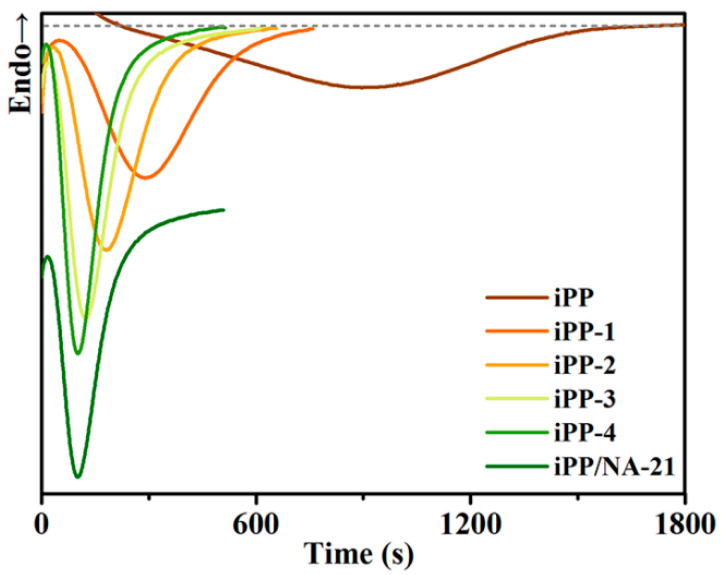
The isothermal crystallization curves for the iPP/A12C-Li composites.

In order to study the synergistic nucleation efficiency between APAl-12C and lilaurate on iPP, the isothermal crystallization behavior for the iPP/A12C-Li composites was investigated under a crystallization temperature of 128 °C, and the corresponding curves are presented in Figure 6. The effect of APAl-12C/Lilaurate concentration on the crystallization rate can be observed clearly and the addition of only 0.05 wt% APAl-12C/lilaurate can shorten the crystallization time from nearly 30 min to about 12 min. As the APAl-12C/lilaurate concentration increased, the crystallization peak became very narrow and sharp, which indicates that the crystallization time is shorter and the crystallization rate has increased rapidly. In addition, it can be seen from Figure 6 that the iPP-4 composite with the addition of 0.5 wt% APAl-12C/lilaurate has almost the same crystallization time compared with that of the iPP/NA-21 composite with 0.5 wt% NA-21, which further proves that the crystallization effect of APAl-12C/lilaurate on the iPP is equivalent to that of NA-21.

In order to further evaluate the nucleation and crystal growth of the iPP/A12C-Li composites, the classical Avrami model can be used to analyze the crystallization kinetics as in the following, Equation (1) [37,38]:

(1) $$1-X_{t}=\exp\left(-Kt^{n}\right)$$

where the Avrami exponent *n* is relative to the nucleation mechanism and growth manner of the crystal, the crystallization rate constant *K* depends on the crystallization temperature and contains both nucleation and growth contributions, and the relative crystallinity (*X_t_*) is calculated with Equation (2) [39] at a different crystallization time.

The *X_t_* is calculated by the integration of the crystallization peaks at different crystallization times, Equation (2):

(2) $$X_{t}=\frac{X_{t}\left(t\right)}{X_{t}\left(\infty \right)}=\frac{\int_{0}^{t}\left(\frac{dH\left(t\right)}{dt}\right)dt}{\int_{0}^{\infty }(\frac{dH\left(t\right)}{dt})dt}$$

where (*dH* (*t*)/*dt*) represents the heat flow. *X_t_* (*t*) and *X_t_* (∞) denote the absolute crystallinity at time (*t*) and at the termination of the crystallization process, respectively.

The Avrami equation can be written as follows (3):

(3) $$\ln\left[-\ln\left(1-X_{t}\right)\right]=nlnt+lnK$$

The values of semicrystalline time (*t*_1/2_) are generally used to describe the crystallization rate of iPP. The *t*_1/2_^a^ calculated from the Avrami model can be calculated as follows (4) [40]:

(4) $$t_{1/2}={\left(\frac{ln2}{K}\right)}^{1/n}$$

**Figure 7:**
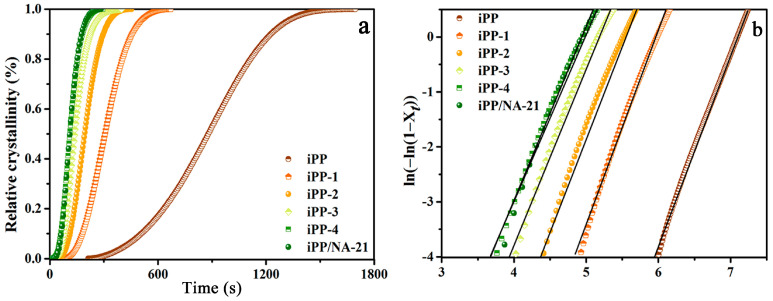
The relative crystallinity curves (**a**) and the relation of ln [−ln (1 − *X_t_*)] versus *lnt* (**b**) for the iPP/A12C-Li composites.

The nucleation effect of APAl-12C/lilaurate is evaluated more evidently by the relative crystallinity curves reported in Figure 7a. It can be seen that all the curves show the S-type characteristic of the polymer material crystals [41]. The relation of ln [−ln (1 − *X_t_*)] versus *lnt* of the iPP/A12C-Li composites is illustrated in Figure 7b, and the calculated values of *n* and *K* are summarized in Table 2. The value of *n* for neat iPP is about 3.60, which is close to the value of 3.70 reported in the literature [42], suggesting that the iPP crystallization mechanism is homogeneous nucleation and three-dimensional spherulite growth. While adding a small amount of APAl-12C/lilaurate, the value of *n* is close to that of neat iPP, indicating that the low content of APAl-12C/lilaurate will not change the spherulite growth patterns of iPP. However, with the increased addition of APAl-12C/lilaurate, the *n* value gradually approaches 3 and the spherulite growth patterns transform from homogeneous nucleation to heterogeneous nucleation. Moreover, the crystallization rate constant’s *K* increases with the addition of APAl-12C/lilaurate, revealing that the increasing of APAl-12C/lilaurate content is beneficial to improving the crystallization rate under the temperature of 128 °C. As we all know, the shorter the *t*_1/2_ value, the faster the crystallization rate [43]. The value of *t*_1/2_^a^ is related to the *n* and *K* parameters, and the calculated values are summarized in Table 2. Meanwhile, the *t*_1/2_^b^ determined from Figure 7a is listed in Table 2, too. There is no significant difference between *t*_1/2_^a^ and *t*_1/2_^b^, which demonstrates further that the Avrami equation is quite successful for analyzing the experimental data of the isothermal crystallization kinetics for the iPP/A12C-Li composites. With the increasing content of APAl-12C/lilaurate, the change trend of *t*_1/2_ is the same as that of the *n* value, and the addition of APAl-12C/lilaurate can shorten *t*_1/2_ of iPP obviously. Under the crystallization temperature of 128 °C, the *t*_1/2_ of neat iPP is about 901.8 s, which is longer than that of the iPP composites with APAl-12C/lilaurate. With the increasing content of APAl-12C/lilaurate, the value *t*_1/2_ decreases sharply. When the addition amount is 0.5 wt%, the *t*_1/2_ is the shortest and reaches 112.8 s, which is more than 7 times shorter than that of pure iPP, indicating better nucleation efficiency and a faster crystallization rate. It can be ascribed to the incorporation of APAl-12C/lilaurate in iPP, which promotes the increase of nucleation sites and the free energy barrier of nucleation is reduced because of the existing epitaxial crystallization, thereby resulting in an increase in the nucleation rate. The *t*_1/2_ value of the iPP composite with 0.5 wt% APAl-12C/lilaurate is the same as that of the iPP composite with 0.5 wt% NA-21, which indicates that the crystallization rate of the APAl-12C/lilaurate is equal to that of NA-21, which further suggests that the crystallization rate of APAl-12C/lilaurate is equal to that of NA-21.

In summary, we can draw the conclusion that the replacement of the hydroxyl group on the APAl-OH structure with lauroyloxy can improve the crystallization ability of iPP to some extent, but the crystallization ability of the iPP/APAl-12C is far inferior to that of iPP/NA-21, which further proves that the direct substitution of the alkyl carboxylic structure cannot achieve the synergistic nucleation of APAl-OH and lithium alkyl carboxylate, and the role of metal Li is irreplaceable in terms of the crystallization behavior of iPP.

### 3.4. The Crystal Morphology for the iPP/A12C-Li Composites

**Figure 8:**
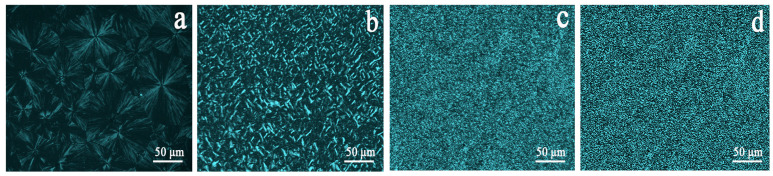
POM micrographs for the iPP/A12C-Li composites under isothermal crystallization temperature of 128 °C: (**a**) iPP; (**b**) iPP-1; (**c**) iPP-3; (**d**) iPP/NA-21.

POM is the most intuitive tool to observe the nucleation and the crystal growth process of the crystalline polymer. Figure 8 exhibits the POM micrographs for the iPP/A12C-Li composites under the isothermal crystallization temperature of 128 °C. Obviously, the neat iPP has a few nuclei and grows in a three-dimensional mode with large spherulites and clear boundaries [44]. The presence of 0.05 wt% APAl-12C/Lilaurate effectively increases the number of nuclei, significantly reduces the grain size and the growth mode is the same as that of the neat iPP. However, when the addition amount is 0.5 wt%, both APAl-12C/lilaurate and NA-21, a large number of nuclei are induced within a short time, accelerating the crystallization of iPP, where the spherulites overlap each other and hence become much smaller than those in pristine iPP. This could be attributed to the presence of the APAl-12C/lilaurate nucleating agent, exhibiting an excellent nucleation effect for the iPP [45].

### 3.5. The Optical Property for the iPP/A12C-Li Composites

**Figure 9:**
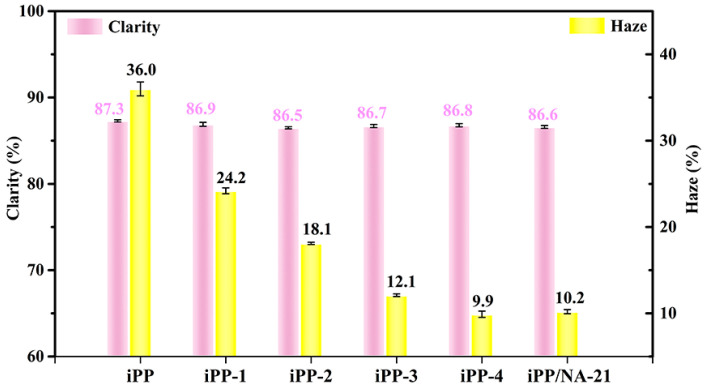
The optical performances for the iPP/A12C-Li composites.

**Figure 10:**
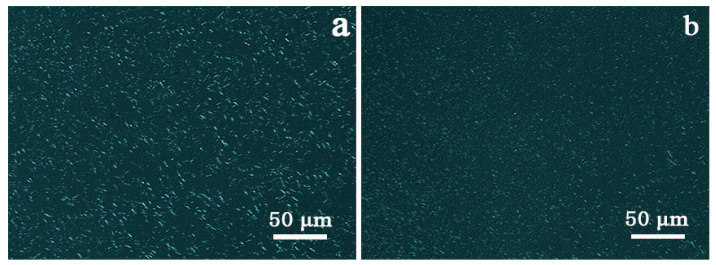
The POM micrographs for iPP-3 (**a**) and iPP/NA-21 (**b**) composites under isothermal crystallization at 135 °C for 5 min.

Thus, we measured the clarity and haze values of the iPP/A12C-Li composites with different concentrations of APAl-12C/lilaurate, and the results are presented in Figure 9. It is fortunate that the addition of APAl-12C/lilaurate will not lead to a change in clarity value. However, the haze value reduces rapidly along with the increase in the concentration of APAl-12C/lilaurate, which is consistent with the crystallization temperature results of the iPP/A12C-Li composites. The haze value is decreased to less than 15% under the concentration of 0.3 wt% APAl-12C/lilaurate. When adding 0.5 wt% APAl-12C/lilaurate to the iPP matrix, the haze value of the iPP composite is only 9.89%, which is lower than that of NA-21 at the same concentration. From this, we can draw the conclusion that the substitution of the lauroyloxy group reduces the overall polarity of APAl-12C/lilaurate, making the dispersion of APAl-12C/lilaurate in the iPP more uniform than NA-21, thus improving the optical properties of the iPP [46]. This conjecture can also be verified from the POM images of the iPP/A12C-Li composite and the iPP/NA-21 composite. As presented in Figure 10, the rod-shaped nuclei in the iPP matrix could be observed clearly. This could be attributed to the heterogeneous nucleation of the nucleating agent. Moreover, the APAl-12C/Lilaurate nuclei disperse uniformly in the iPP matrix. However, the nuclei of the NA-21 are prone to aggregation, indicating that the dispersion of NA-21 in the iPP matrix is underdeveloped, which is further explained by SEM of the iPP composites.

### 3.6. The Dispersion of Nucleating Agents for the iPP/A12C-Li Composites

**Figure 11:**
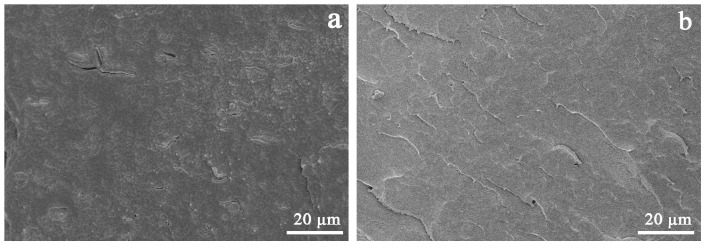
The SEM micrographs of the iPP/NA-21 composite (**a**) and iPP-3 composite (**b**).

The interfacial interaction and dispersion of the nucleating agent in polymers has a great influence on application properties, such as crystallization and mechanical and optical properties [47]. In order to compare the dispersion of APAl-12C/lilaurate and NA-21 in the iPP matrix, the fracture surface of the iPP/A12C-Li composites is observed using SEM. As described in Figure 11a, the NA-21 particles are dispersed unevenly in the iPP matrix. Moreover, some agglomeration and gaps can be observed, which indicates the poor interfacial interaction between NA-21 and iPP. On the contrary, there are no obvious visible particles in the iPP-3 composite with 0.5 wt% APAl-12C/lilaurate in Figure 11b, which suggests that APAl-12C/lilaurate is uniformly dispersed in the iPP matrix. The SEM result demonstrates that APAl-12C/lilaurate has better compatibility with the iPP than that of the NA-21, which owes to the fact that the replacement of the lauroyloxy for hydroxyl enhances the interfacial interaction between the APAl-12C/lilaurate and the iPP matrix, enhancing the application properties of the iPP in turn. The SEM results are consistent with the conclusion of the POM.

### 3.7. The Mechanical Property for the iPP/A12C-Li Composites

**Figure 12:**
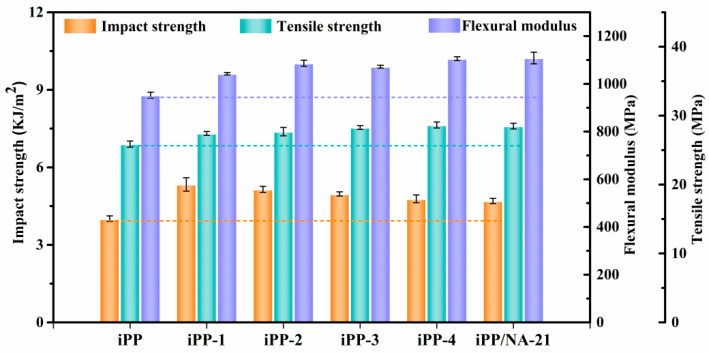
The mechanical properties for the iPP/A12C-Li composites.

The study of the effects of APAl-12C/lilaurate with different contents on the mechanical properties of the iPP has an instructive significance for commercial application. Figure 12 shows the mechanical performances of the iPP/A12C-Li composites, and the full outcomes are summarized in Table 3. During the addition of APAl-12C/lilaurate, the flexural modulus and tensile strength of the iPP composites were enhanced gradually. When the addition amount reached 0.5 wt%, both the flexural modulus and tensile strength reached their maximum values, which were increased by 16.3% and 10.6%, respectively. The tensile strength and the flexural modulus values demonstrate an identical tendency. The toughness and rigidity generally displayed the opposite tendency. The addition of the APAl-12C/lilaurate enhanced the impact strength of the iPP. When the addition amount was 0.05 wt%, the impact strength rose most significantly, which enhanced by 33.2%. However, the change in the impact strength is the opposite to the change in addition amount of APAl-12C/lilaurate. With the increase in addition amounts of APAl-12C/lilaurate, the impact strength decreased gradually, and still increased by about 19.2% when the addition amount was 0.5 wt%. Furthermore, there are no significant differences observed in the tensile strength, flexural modulus and impact strength results with the nucleating agent of APAl-12C/lilaurate and NA-21 in the iPP. It can be noted that the incorporation of APAl-12C/lilaurate and NA-21 has a considerable influence on the mechanical properties and has an exceptional equilibrium on the rigidity and toughness.

## 4. Conclusions

The APAl-12C nucleating agent was synthesized by lauroyloxy, which substituted the hydroxyl in APAl-OH. The nucleation ability of the APAl-12C for iPP was analyzed by DSC and POM. The T_c,p_ of the iPP/APAl-12C composites is significantly lower than that of iPP/NA-21 (NA-21 is compounded with APAl-OH and lithium alkylate), which can be explained by the fact that the direct substitution of the alkyl carboxylic structure cannot achieve synergistic nucleation of the APAl-OH and lithium alkyl carboxylate for iPP. When lithium laurate and APAl-12C are added into the iPP matrix, it is found that the T_c.p_ for iPP is greatly improved by the APAl-12C/lilaurate nucleating agent, which is basically equivalent to that of the NA-21 under the same concentration. This result also proves that Li metal is an indispensable component in terms of crystallization performance. With the increase in the APAl-12C/lilaurate amounts, the crystal morphology of the iPP/A12C-Li composites changed from three-dimensional spherulite to two-dimensional growth mode, and the *n* value of 0.5 wt% APAl-12C/lilaurate was close to 2. The tensile strength, flexural modulus and impact strength of the iPP/A12C-Li composites with 0.5 wt% of APAl-12C/lilaurate increased by 10%, 16% and 19%, respectively. The presence of APAl-12C/lilaurate not only increases the rigidity but also improves the toughness of iPP, realizing the balance of rigidity and toughness of iPP, which is due to the weakening of the polarity of APAl-12C after lauroyloxy substitution and better dispersion in the iPP matrix, resulting in significant improvement in the mechanical properties. Moreover, the increase in molecular weight of the nucleating agent had an important influence on reducing the migration potential in the iPP matrix, lowering the impact on the environment.

## Figures and Tables

**Table 1:** The formula of the iPP/A12C-Li composites.

Samples	iPP/wt%	APAl-12C/Lilaurate (6:4, Mass Ratio)/wt%
iPP-1	99.95	0.05
iPP-2	99.90	0.10
iPP-3	99.70	0.30
iPP-4	99.50	0.50
iPP/NA-21	99.50	0.50

**Table 2:** The isothermal crystallization parameters for the iPP/A12C-Li composites.

Samples	*T* (°C)	*n*	*K*	*t*_1/2_ ^a^ (s)	*t*_1/2_ ^b^ (s)
iPP	128	3.60	0.00004	900.6	901.8
iPP-1		3.70	0.00160	309.6	307.2
iPP-2		3.52	0.01045	197.4	195.6
iPP-3		3.38	0.03802	141.6	139.2
iPP-4		3.27	0.08278	114.6	112.8
iPP/NA-21		3.48	0.07364	114.6	112.2

^a^ Calculated from Avrami parameters. ^b^ Determined from Figure 7a.

**Table 3:** The mechanical performances for the iPP/A12C-Li composites.

Sample	Impact Strength (KJ/m^2^)	Tensile Strength (MPa)	Flexural Modulus (MPa)
iPP	4.0 ± 0.11	25.9 ± 0.45	952.7 ± 12.48
iPP-1	5.3 ± 0.26	27.4 ± 0.28	1042.6 ± 5.32
iPP-2	5.2 ± 0.12	27.7 ± 0.61	1086.6 ± 12.83
iPP-3	4.0 ± 0.08	28.3 ± 0.28	1072.1 ± 5.89
iPP-4	4.8 ± 0.15	28.7 ± 0.43	1105.8 ± 8.03
iPP/NA-21	4.7 ± 0.10	28.5 ± 0.41	1108.5 ± 24.29

## Data Availability

Not applicable.
